# The Wellbeing of Italian Peacekeeper Military: Psychological Resources, Quality of Life and Internalizing Symptoms

**DOI:** 10.3389/fpsyg.2018.00103

**Published:** 2018-02-13

**Authors:** Yura Loscalzo, Marco Giannini, Alessio Gori, Annamaria Di Fabio

**Affiliations:** ^1^Department of Health Sciences, School of Psychology, University of Florence, Florence, Italy; ^2^Department of Human Sciences, LUMSA University, Rome, Italy; ^3^Psychology Section, Department of Education and Psychology, University of Florence, Florence, Italy

**Keywords:** military psychiatry, peacekeeping operations, psychological resources, PTSD, internalizing symptoms, quality of life

## Abstract

Working as a peacekeeper is associated with the exposure to acute and/or catastrophic events and chronic stressors. Hence, the meager literature about peacekeepers’ wellbeing has mainly analyzed Post-Traumatic Stress Disorder (PTSD). This study aims to deep the analysis of the wellbeing of peacekeepers military. Based on the few studies on this population, we hypothesized that Italian peacekeeper military officers and enlisted men (*n* = 167; 103 males, 6 females, 58 missing) exhibit lower levels of internalizing symptoms (i.e., PTSD, depression, general anxiety, obsessions, and somatization) as compared to a control group (*n* = 60; 32 males, 28 females). Moreover, we hypothesized that peacekeepers have higher levels of psychological resources (i.e., self-efficacy, self-esteem, social support) and quality of life (i.e., higher life satisfaction and lower general stress). We compared the groups by means of MANOVAs on the subscales of the Psychological Treatment Inventory (PTI; Gori et al., 2015). We found that Italian peacekeepers have lower internalizing symptoms and higher levels of self-efficacy and self-esteem than the control group; however, no statistically significant differences were observed on perceived social support. Finally, peacekeepers have a higher quality of life: scores reflect higher life satisfaction and lower distress than the control group. This study is in line with previous literature supporting the claim that Italian peacekeeper military officers have sufficient psychological resources for coping with the stressful situations implied in peacekeeping missions. Future studies should deepen the analysis of the military’s psychological characteristics by comparing war veterans and peacekeeper military.

## Introduction

In the Cold War era, after a peace treaty was signed, unarmed or lightly armed military personnel acted as a buffer between warring factions. However, since the late 1980s, following the collapse of Cold War and an increase in conflicts worldwide, Peacekeeping Operations (PKOs) grew in both size and complexity (Raju, 2014). PKOs became more and more hybrid since they were characterized by modular and multi-actor actions. They employ many kinds of military and civilian activities, at the same time working with multiple different institutions, in order to favor peacekeeping and peacebuilding (Tardy, 2014). Peace operations cover conflict prevention, peacekeeping, and peacebuilding (Smith, 2016). Accordingly, the High-Level Independent Panel on Peace Operations (HIPPO; United Nations, 2015) stressed that PKOs make use of many different tools, such as special envoys and mediators, and address many different scenarios, such as political missions, observation missions that include both ceasefire and electoral missions, or technical specialist missions. Though, it should be noted that PKOs have become more and more militarized, as the vision of peace has been joined to the means of violence (Pugh, 2012; Suhrke, 2012). Military violence in PKOs is an issue that has to be taken into account, even if it is rarely problematized and usually associated with the conflict belligerence only. Peacekeepers’ violence is seen instead as peace operations for mitigating and deterring the violence of the belligerents (Charbonneau, 2012). In line with this, Kühn (2012) stressed that the term ‘peace’ has changed its real meaning in a virtual term that sometimes covers violent management techniques.

Because of this complexity, peacekeepers face a diversity of stressors that vary between missions related to the level of difficulty, the length of the mission, or the local culture (Shigemura and Nomura, 2002), and stressors common across missions. These common stressors can come from pre-deployment challenges, such as demands for adjustment due to the possibility of combat, uncertainty of the duration of the engagement, or anticipation of difficulties in communication with family. There are also deployment stressors. As for example, they can experience physical and psychological isolation and role conflict. Peacekeepers are trained for war, but in PKOs they are requested to maintain peace following strict rules of engagement. Moreover, they can also experience boredom and witness atrocities and they are exposed to dangers, such as land mines or endemic diseases. Finally, peacekeeping soldiers commonly experience also post-deployment stressors, such as guilt, reworking relationships, and changes in emotionality (cf. Raju, 2014).

Given the many stressors faced by peacekeeper soldiers, some scholars analyzed the wellbeing of these workers. While all working environments are associated with some stress (e.g., Shaw et al., 2012; Halouani et al., 2017) working as a peacekeeper may be one of the most stressful jobs (Keskinen and Simola, 2015).

Even if peacekeepers usually face situations characterized by lower stress as compared to traditional combat situations, such as the Vietnam War, they are exposed to stress as well. Lamerson and Kelloway (1996) stated that peacekeepers deal with both acute and/or catastrophic events and chronic stressors. More specifically, acute stressors are events characterized by a specific time of onset, high intensity, and low frequency of occurrence (such as being fired), while catastrophic events are also characterized by threat to life and/or involve larger groups as a community and cause prolonged suffering (besides the three acute stressors’ features). Finally, chronic stressors have no specific time of onset and frequent occurrence, and they may have either low or high intensity.

Given the many stressors faced by peacekeepers, it is not surprising that the majority of studies about these workers’ wellbeing analyzed the Post-traumatic Stress Disorder (PTSD).

The PTSD is defined by the Diagnostic and Statistical Manual of Mental Disorder (DSM-5; American Psychiatric Association [APA], 2013) as a clinical disorder that develops after the exposure to a traumatic event, such as the exposure to an actual or threatened death or sexual violence, both by means of a direct or indirect (e.g., witnessing in person the event) experience of the event. It is characterized by at least one of the following symptoms: intrusive memories or dreams about the event, dissociation (e.g., flashbacks), distress following the exposure to internal or external cues related to the traumatic event, and marked physiological reactions to these cues. Moreover, the person avoids the stimuli associated to the traumatic event, there are some alterations in cognition (e.g., not being able to remember important aspects of the event) and mood (e.g., inability to experience positive emotions), and marked alterations in arousal and reactivity linked to the event.

The studies on peacekeepers’ health showed that some develop PSTD, despite many of them adjust well to the stressors experienced during the deployment; they do not develop neither PSTD nor other disorders (Weisaeth et al., 1993; MacDonald et al., 1996; Litz et al., 1997; Orsillo et al., 1998; Ismail et al., 2002; Mehlum and Weisaeth, 2002; Brett et al., 2003; Hotopf et al., 2003a; Michel et al., 2003; Klaassens et al., 2008; Sareen et al., 2008).

As far as concern other internalizing symptoms besides the PSTD ones (i.e., inner and covert behaviors such as rumination) and externalizing symptoms (i.e., overt behaviors such as aggressiveness), Klaassens et al. (2008) showed that the 83% of the Dutch veteran peacekeepers that participated in their research did not report significant psychological distress about 10 and 25 years after deployment. The 17% of peacekeepers scored instead above the cut-off of the instrument they used, which allows evaluating both internalizing (somatic complaints, cognitive problems, interpersonal sensitivity, depression, anxiety, phobic fear) and externalizing (hostility, paranoid thinking, psychoticism) symptoms. The percentage of peacekeepers with psychological problems, besides being small as compared to the percentage of not-impaired peacekeepers, is also equal to a Dutch norm group. The only group-difference found by Klaassens et al. (2008) concerns hostility, as peacekeepers have more hostility than the control group. In addition, also Chinese medical peacekeepers had a good mental health status 1 week after their arrival in Lebanon (Li et al., 2012). In line with these studies, Hotopf et al. (2003a) did not find higher risk for anxiety and depression symptoms in British peacekeepers.

Suicide is another mental health-related factor that has been studied more recently. The studies on this topic showed that the increased risk of suicide in peacekeepers veterans is higher in the ones who have prematurely repatriated (Ponteva et al., 2000; Thoresen et al., 2003, 2006; Thoresen and Mehlum, 2008). Moreover, mental health problems, living alone, and the break-up of a love relationship are suicide risk factors, while peacekeeping-related factors are not (Thoresen and Mehlum, 2006).

Finally, it is interesting to note that Proctor et al. (2009) highlighted that US peacekeepers who have been deployed, as compared to non-deployed soldiers, showed reduced proficiency in motor speed and sustained attention tasks, but also an improved proficiency in working memory tasks and fewer depression symptoms, about 7 months after deployment. Hence, these peacekeepers showed an adaptive response to the stressors faced during deployment, both as far as concern neuropsychological functioning and mood.

If the literature about peacekeepers’ mental health is meager, the studies about their psychological resources (i.e., self-efficacy, self-esteem, social support) and quality of life (i.e., life satisfaction and general stress) are even less.

Concerning psychological resources, to the best of our knowledge, there is only the study by Dirkzwager et al. (2003) that, however, analyzed social support and coping strategies as PTSD predictors among Dutch peacekeepers. The results showed that fewer and more negative social contacts are associated with higher PTSD symptoms severity. In addition, the coping strategies associated to higher symptoms are the wishful thinking (i.e., the creation of beliefs according to desires and not accordingly to evidence and rationality) and accepting responsibility (i.e., over-evaluating one’s contribution to the traumatic event, with following feelings of guilt) strategies, while planful problem solving and seeking social supports are associated with less PTSD symptoms.

Moreover, as far as we know, there is only one study addressing stress as an outcome of PKOs, and it showed that Italian peacekeepers did not have an increase in their self-report stress (Ballone et al., 2000). Hotopf et al. (2003b) analyzed instead demographic and peacekeeping related factor as predictors of stress.

In sum, the few studies about peacekeepers’ health support the notion that the large majority of soldiers do not develop internalizing psychopathology, as for example PTSD (e.g., Ismail et al., 2002; Brett et al., 2003; Klaassens et al., 2008; Sareen et al., 2008). These results are in line with the studies that highlight that peacekeepers generally perceive their missions in a positive vein: the missions improve technical skills, self-efficacy and ability to cope with stress, and help them to feel useful (Mehlum, 1995; Weisaeth et al., 1996).

Since the literature on peacekeepers’ wellbeing is limited, we aim to deepen the analysis of this aspect on Italian military peacekeepers. More specifically, we evaluate PTSD, as it is the most analyzed disorder in peacekeeper literature. Then, we analyze other internalizing symptoms since, as they are covert behaviors, they are more difficult to detect as compared to externalizing (overt) symptoms. Though, they are distressing and lead to a functional impairment as well as the externalizing ones. Finally, given that there are few studies on quality of life and psychological resources, we also aim to address this gap by analyzing them in the present research.

Based on the few studies on psychological wellbeing on peacekeepers, we hypothesize that Italian peacekeepers differ from a control group according to several factors: (i) peacekeepers have lower levels of PSTD symptoms (Weisaeth et al., 1993; MacDonald et al., 1996; Litz et al., 1997; Orsillo et al., 1998; Ismail et al., 2002; Mehlum and Weisaeth, 2002; Brett et al., 2003; Hotopf et al., 2003a; Michel et al., 2003; Klaassens et al., 2008; Sareen et al., 2008); (ii) peacekeepers have lower levels of other internalizing symptoms, namely depression, general anxiety, obsessions and somatization symptoms (Hotopf et al., 2003a; Michel et al., 2003; Klaassens et al., 2008; Proctor et al., 2009; Li et al., 2012); (iii) peacekeepers have higher psychological resources that buffer the stress they experience (Mehlum, 1995; Weisaeth et al., 1996). More specifically, they have higher self-efficacy, self-esteem and perceived social support; (iv) as a consequence of (i), (ii), and (iii), peacekeepers report a higher quality of life, or they have higher life satisfaction and lower distress (Ballone et al., 2000).

The results of this study could help to develop preventive interventions (e.g., Di Fabio, 2016; Di Fabio and Kenny, 2016a,b; Di Fabio et al., 2016), such as selecting for peacekeeping missions soldiers with enough psychological resources (e.g., Di Fabio, 2014, 2015; Di Fabio and Saklofske, 2014; Di Fabio and Kenny, 2015; Di Fabio et al., 2017a) for coping with the associated stressors, and hence sustaining an healthy organization for military peacekeepers (Di Fabio, 2017a,b; Di Fabio et al., 2017b). Moreover, this study could suggest which psychological resources should be strengthened in order to avoid psychological maladjustment of soldiers engaged in peacekeeping missions.

## Materials and Methods

### Participants

We recruited 227 participants: 167 peacekeepers (73.6% of the sample) and 60 subjects as the control group (26.4%).

Peacekeeper military officers were only selected if they were involved in a peacekeeping mission. Some of them did not provide their sociodemographic characteristics. Based on the available data, they were aged between 20 and 52 years (*M* = 31.70, *SD* = 7.52, *n* = 105), and were mostly males (61.7%, *n* = 103). There were few females (3.6%), and 34.7% participants did not report their gender. Concerning their education, the majority done a secondary school of second level (43.7%), while 13.2% completed the secondary school of first level, a few hold a master degree (4.8%), and 38.3% peacekeepers did not report their education level.

Finally, there were 39.5% enlisted men, 13.8% non-commissioned (or warrant) officers, and 2.4% commissioned officers (44.3% participants did not provide this information). It should be noted that such different rank in the Army are associated with different roles, and hence to varying degree and different kinds of stress. Enlisted soldiers might be defined as the backbone of the Army, as they have specific and operative tasks; hence, they are the workforce. Officers are instead the managers of the enlisted men, as they plan missions, they assign them to specific tasks, and they give them orders. Among officers, there is a distinction between non-commissioned (or warrant) officers and commissioned officers. Commissioned officers give orders and assign tasks to all the personnel, including non-commissioned officers. Non-commissioned officers give instead orders to enlisted man only and, by means of these orders, they have to translate the commissioned officers’ directions into instructions for the enlisted men with the aim of completing the job requested by the commissioned officers. They have a focused and technical expertise (United States Army, 2017).

Based on these definitions, it follows that while enlisted men have higher levels of stress associated to life-threatening events; officers have instead higher levels of stress associated to their management role and their responsibility for lower-ranked army soldiers.

The participants of the control group were 53.5% males and 46.7% females aged between 20 and 64 years (*M* = 30.67, *SD* = 9.66). With respect to their educational level, 18% participants completed the secondary school of first level, 65% had done a secondary school of second level, and a few held a master degree (17%).

### Materials

We administered some scales of the Psychological Treatment Inventory (PTI; Gori et al., 2015). The PTI has two different forms: a patient, or self-report version, and a form filled out by a clinician who knows him/her – the clinician version. In this study, we used some scales of the participant version only, which have to be filled by the participants by means of a 5-point scale (1 = not at all, 2 = somewhat, 3 = moderately, 4 = a good deal, and 5 = very much).

More specifically, for assessing the internalizing symptomatology, we administered the following scales: Post-traumatic stress disorder (e.g., The memory of a shocking experience that I have experienced is still tormenting me), Depression (e.g., I’ve lost interest in things I do), General anxiety (e.g., I feel anxious), Obsessions (e.g., I have difficulty concentrating), and Somatization (e.g., My shoulders and neck muscles are often stiffened, contracted). In addition, for evaluating the Psychological Resources, we administered the following scales: Self-efficacy (e.g., I am able to exploit my skills in a productive way); Self-esteem (e.g., I’m an interesting person); and Perceived social support (e.g., When I’m in trouble, I know I can rely on someone’s help). Finally, in order to evaluate the Quality of Life, we administered the Life satisfaction (e.g., Overall, I’m happy with my life) and the Distress (e.g., At the end of the day I feel exhausted) scales.

The PTI has good psychometric properties; α coefficients indicate good internal consistency for all scales, except for those examining profile validity. Test–retest reliabilities are also good, ranging from 0.75 to 0.95 (Gori et al., 2015). In the present research, the values of Cronbach’s alpha are: Post-traumatic stress disorder, α = 0.75; Depression, α = 0.79; General anxiety, α = 0.86; Obsession, α = 0.83; Somatization, α = 0.80; Self-efficacy, α = 0.85; Self-esteem, α = 0.71; Perceived Social Support, α = 0.75; Life satisfaction, α = 0.78; Distress, α = 0.78.

### Procedure

First, we asked for the authorization to the Department of Health Sciences of the University of Florence. Next, we asked for the authorization to conduct the research to the Staff of Defense, by means of their Communication Office.

Once we get the authorization, we gave the questionnaires to the Communication Office, which helped us in the administration of the questionnaire to the soldiers who were in service. The Communication Office organized the administration in a collective form and in a quiet place. Before filling the questionnaire, the participants were provided with the Informed Consent, which they had to sign in order to take part in the research.

The questionnaire comprehended the PTI, preceded by some demographic questions (gender, age, education level). Although assurances were given that data would be kept anonymous, some of the participants did not report these data.

### Data Analysis

We conducted the statistical analysis using the software SPSS.20. Three hypotheses were tested by means of three MANOVAs; internalizing symptoms, psychological resources, and quality of life were analyzed separately. Since many participants belonging to the peacekeeper group did not provide their demographics (i.e., gender, age, education level) and rank level (i.e., enlisted man, non-commissioned officer or commissioned officer), we did not control for these variables in the MANOVAs we performed in order to avoid a pronounced reduction of the number of participants.

The preliminary analyses that we done in order to check the normality assumption showed that some of the variables (i.e., the scales evaluating internalizing symptoms) have values of kurtosis and skewness that do not meet the ±1 cut-off criteria (see **Table 1**). Moreover, the Box’s Test of Equality of Covariance Matrices showed that the data violate the assumption of homogeneity of variance–covariance matrices for the MANOVA we conducted with the internalizing scales, and the Levene’s Test of Equality of Error Covariance showed that the related assumption is violated for the Somatization scale. However, for all the other internalizing scales the assumption is met. About the MANOVAs with psychological resources and quality of life scales as dependent variables, the two above-mentioned assumptions are met instead.

**Table 1:** Kurtosis and Skewness values for the dependent variables (*n* = 227).

PTI variable	*M* (*SD*)	Kurtosis	Skewness
Self-efficacy	25.05 (4.97)	-0.04	-0.56
Self-esteem	19.85 (4.00)	0.07	-0.19
Perceived social support	20.19 (4.28)	-0.21	-0.03
Depression	9.68 (3.67)	1.59	1.93
General anxiety	10.48 (4.50)	1.65	2.68
Obsessions	9.05 (3.77)	1.37	1.11
Somatization	8.95 (3.99)	1.54	1.82
Post-traumatic stress disorder	10.41 (4.04)	1.30	1.10
Life satisfaction	18.63 (4.15)	-0.39	-0.60
Distress	8.13 (3.08)	1.04	0.50

*PTI, Psychological Treatment Inventory.*

In conclusion, the normality and homoscedasticity assumptions are violated for the MANOVA related to the internalizing symptoms only. We performed the MANOVA anyway, as it “is reasonably robust to moderate violations of normality” (Pallant, 2001, p. 219), especially if there are at least 20 subjects in each cell (Tabachnick and Fidell, 1996) or, even better, over 30, as in this case any violations of normality or equality of variance does not matter too much (Pallant, 2001). Hence, given that the sample size of our groups is well above 30 for both the peacekeepers and the control groups, we proceeded with MANOVAs analysis despite the normality assumption violations for some of the dependent variables.

## Results

### PTSD and Other Internalizing Symptoms

For testing hypotheses 1 and 2, namely that peacekeepers have lower levels of PTSD symptoms or other internalizing symptoms; we performed a MANOVA with the following scales of the PTI as dependent variables: Post-traumatic stress disorder, Depression, General anxiety, Obsessions, and Somatization.

The multivariate test showed a statistically significant effect: *F*(5,221) = 12.81, *p* < 0.001, $\eta_{p}^{2}$ = 0.23. Subsequent ANOVAs showed that the peacekeeper group has significantly lower symptoms of Post-Traumatic Stress Disorder, *F*(1,225) = 17.88, *p* < 0.001, $\eta_{p}^{2}$ = 0.07, Depression, *F*(1,225) = 25.73, *p* < 0.001, $\eta_{p}^{2}$ = 0.10, General Anxiety, *F*(1,225) = 40.96, *p* < 0.001, $\eta_{p}^{2}$ = 0.15, Obsessions, *F*(1,225) = 51.65, *p* < 0.001, $\eta_{p}^{2}$ = 0.19, and Somatization, *F*(1,225) = 34.41, *p* < 0.001, $\eta_{p}^{2}$ = 0.13, (see **Table 2** for the descriptive statistics).

**Table 2:** Means (SDs) of the internalizing symptoms by peacekeepers and control group.

PTI variable	Group	*M* (*SD*)	*n*
Depression	Peacekeepers	8.98 (3.33)	167
	Control	11.63 (3.88)	60
	Total	9.68 (3.67)	227
General anxiety	Peacekeepers	9.43 (3.94)	167
	Control	13.42 (4.67)	60
	Total	10.48 (4.50)	227
Obsessions	Peacekeepers	8.08 (3.39)	167
	Control	11.77 (3.48)	60
	Total	9.05 (3.77)	227
Somatization	Peacekeepers	8.08 (3.50)	167
	Control	11.37 (4.29)	60
	Total	8.95 (3.99)	227
Post-traumatic stress disorder	Peacekeepers	9.75 (3.88)	167
	Control	12.23 (3.93)	60
	Total	10.41 (4.04)	227

*PTI, Psychological Treatment Inventory.*

### Psychological Resources

Next, in order to test the third hypothesis, that peacekeepers have higher psychological resources, we conduct a MANOVA with the following psychological resources as dependent variables: self-efficacy, self-esteem, and perceived social support.

The multivariate test showed a statistically significant effect: *F*(3,223) = 8.38, *p* < 0.001, $\eta_{p}^{2}$ = 0.10. Subsequent ANOVAs showed that the military group has significantly higher self-efficacy, *F*(1,227) = 25.06, *p* < 0.001, $\eta_{p}^{2}$ = 0.10 and self-esteem, *F*(1,227) = 10.41, *p* = 0.001, $\eta_{p}^{2}$ = 0.04, than the control group. There is not difference on perceived social support, *F*(1,227) = 3.21, *p* = 0.08, $\eta_{p}^{2}$ = 0.01 (see **Table 3** for the descriptive statistics).

**Table 3:** Means (SDs) of the psychological resources by peacekeepers and control group.

PTI variable	Group	*M* (*SD*)	*n*
Self-efficacy	Peacekeepers	25.99 (4.72)	167
	Control	22.43 (4.75)	60
	Total	25.05 (4.97)	227
Self-esteem	Peacekeepers	20.35 (3.88)	167
	Control	18.45 (4.04)	60
	Total	19.85 (4.00)	227
Perceived social support	Peacekeepers	20.50 (4.30)	167
	Control	19.35 (4.14)	60
	Total	20.19 (4.28)	227

*PTI, Psychological Treatment Inventory.*

### Quality of Life

Finally, in order to test the last hypothesis, that peacekeeper military officers have a higher quality of life, we done a further MANOVA with Life satisfaction and Distress as dependent variables.

The multivariate test showed a statistically significant effect: *F*(2,224) = 26.29, *p* < 0.001, $\eta_{p}^{2}$ = 0.19. Subsequent ANOVAs showed that the military group has significantly higher level of life satisfaction, *F*(1,227) = 29.74, *p* < 0.001, $\eta_{p}^{2}$ = 0.12, and lower level of distress, *F*(1,227) = 42.73, *p* < 0.001, $\eta_{p}^{2}$ = 0.16, than the control group (see **Table 4** for the descriptive statistics).

**Table 4:** Means (SDs) of the quality of life by peacekeepers and control group.

PTI variable	Group	*M* (*SD*)	*n*
Life satisfaction	Peacekeepers	19.47 (3.90)	167
	Control	16.27 (3.92)	60
	Total	18.63 (4.15)	227
Distress	Peacekeepers	7.40 (2.72)	167
	Control	10.18 (3.12)	60
	Total	8.13 (3.08)	227

*PTI, Psychological Treatment Inventory.*

## Discussion

This study aimed to analyze if low internalizing symptomatology and high psychological resources and quality of life characterize Italian peacekeepers, as suggested by the few studies on this topic (e.g., Mehlum, 1995; Weisaeth et al., 1996; Brett et al., 2003; Klaassens et al., 2008). While stress is present in almost all working environments, working as a peacekeeper may be among the most stressful (Keskinen and Simola, 2015). However, peacekeepers are selected based on a psychological assessment and they are trained for stressful situations; hence, we hypothesized that they have positive characteristics that buffer the stress experienced during missions.

We found that peacekeepers have lower PTSD symptoms than the control group, in line with previous studies (e.g., Brett et al., 2003; Klaassens et al., 2008). We extended the literature on this topic by analyzing other internalizing symptoms, namely depression, general anxiety, obsessions, and somatization symptoms. We hypothesized that the participants were selected for the mission since they were characterized by a low symptomatology. In line with this, we found significantly lower levels for all the internalizing symptomatology we analyzed.

With respect to psychological resources, we found, in line with Mehlum (1995) and Weisaeth et al. (1996), that peacekeepers have higher self-efficacy and self-esteem than the control group. These two resources are particularly important for military officers since they avoid a procrastination of the tasks and they favor a high commitment in the actions required by the missions. Hence, we can assume that peacekeepers have been selected based on their high level of psychological resources. However, they do not exhibit higher levels of perceived social support than the control group. They also do not report a lower level of perceived social support, in spite of being far from their country. This may be because missions were conducted with colleagues who offer the social support otherwise removed by being away from home. Perceived social support is important because it could prevent their psychological health impairment.

Finally, with respect to quality of life, in line with Ballone et al. (2000), we found higher reported quality of life among peacekeepers. They reported higher life satisfaction and lower distress as compared to the control group. We could explain this finding referring to the previous results: peacekeepers have higher psychological resources and lower internalizing symptomatology; consequently, they also have a higher quality of life. One question was whether these results could be accounted for based on a social desirability bias. The participants could have presented themselves in a positive vein for the fear of being identified and maybe fired out. Even though assurances were given that answers would be anonymous, we have to consider that they filled the questionnaire during their mission and in their place of work and that military personnel administered the questionnaires.

A limitation of the present study is the lack of demographic data of many participants, which do not allow to perform group differences analyses, such as between males and females, or between soldiers with different ranks (i.e., between enlisted men and non-commissioned officers), neither to control for their effect when testing group differences by means of the MANOVAs. Moreover, the control group, even if comprehends 60 participants (i.e., more than the minimum of 30 cases recommended by Pallant, 2001 for performing MANOVA analysis), is smaller as compared to the peacekeeper group. Finally, our study is cross-sectional, and hence it does not allow proposing any casual relationships.

Despite these limitations, this study highlights the positive consequences of selecting peacekeepers based in part on their psychological resources to cope with stressful situations. We found low internalizing symptomatology and higher self-esteem, self-efficacy and quality of life in the military as compared to the control group. The study deepens our knowledge about the psychological characteristics of peacekeepers, which is a field understudied. Our findings suggest that it is valuable to select personnel who have military competences, but also high psychological resources that help them to cope with the stressors implied in their mission. Moreover, it would be useful to strengthen their perceived social support, given that it is an important resource.

Future studies should analyze further these psychological characteristics in peacekeeper military, especially by means of a comparison between war veterans and peacekeepers, in order to understand if there are some differences between peacekeeper and the military involved in the war.

## Ethics Statement

This study was carried out in accordance with the recommendations of the Staff of Defense with written informed consent from all subjects. All subjects gave written informed consent in accordance with the Declaration of Helsinki. The protocol was approved by the Staff of Defense.

## Author Contributions

YL managed the literature search, performed the statistical analysis, and drafted the manuscript. MG and AD designed the work. YL, MG, AG, and AD interpreted the data and critically revised the intellectual content of the manuscript. All the authors approved the final version of the manuscript and agreed to be accountable for all aspects of the work in ensuring that questions related to the accuracy or integrity of any part of the work are appropriately investigated and resolved.

## Conflict of Interest Statement

The authors declare that the research was conducted in the absence of any commercial or financial relationships that could be construed as a potential conflict of interest.
